# A Quantitative Exploration of the Relationship Between Healthcare Accessibility and Mass Media in Nigeria Using the Levesque Framework of Healthcare Access

**DOI:** 10.3390/vaccines13090981

**Published:** 2025-09-18

**Authors:** Chelsea Gordon, Teresa Paslawski, Thilina Bandara, Shannon Floer, Tayyab Shah

**Affiliations:** 1 Department of Interdisciplinary Studies, College of Graduate and Postdoctoral Studies, University of Saskatchewan, Saskatoon, SK S7N 5A2, Canada; 2 School of Rehabilitation Sciences, College of Medicine, University of Saskatchewan, Saskatoon, SK S7N 5A2, Canada; teresa.paslawski@usask.ca; 3 School of Public Health, University of Saskatchewan, Saskatoon, SK S7N 5A2, Canada; thilina.bandara@usask.ca; 4 Department of Educational Administration, College of Education, University of Saskatchewan, Saskatoon, SK S7N 5A2, Canada; shannon.floer@usask.ca; 5 Canadian Hub of Applied Social Research, University of Saskatchewan, Saskatoon, SK S7N 5A2, Canada; tayyab.shah@usask.ca

**Keywords:** healthcare, immunization, Nigeria, media, ICT, health literacy, geospatial analysis

## Abstract

**Background/Objectives**: This study investigates the relationship between maternal media access and childhood immunization status in Nigeria using the Levesque Framework for Healthcare Access. **Methods**: Utilizing data from the 2021 MICS-NICS survey, the study analyzes sociodemographic and media/ICT variables through logistic regression and geospatial mapping. **Results**: The results indicate that region is the strongest predictor of immunization status, with significant disparities in access to media and healthcare services across Nigeria. Television exposure was associated with improved immunization outcomes, while mobile phone ownership was not. **Conclusions**: The findings emphasize the importance of equitable media access and tailored health communication strategies to improve healthcare accessibility. The study highlights the need for region-specific interventions and continued monitoring of media access trends to support universal health coverage goals.

## 1. Introduction

The United Nations [1] considers access to high-quality healthcare services and affordable medicines and vaccines to be crucial indicators to meeting Sustainable Development Goal 3: Global Universal Health Coverage (UHC). Progress toward SDG 3 has stagnated over the last 10 years, leaving an estimated 4.5 billion people, or over half the world’s population, without UHC [2]. In 2021, Nigeria faced one of the lowest coverage rates of UHC and was estimated to have the lowest rates of childhood immunization coverage in the world [2].

The Nigerian government has renewed its commitment to achieving UHC by passing the National Health Insurance Act bill in 2022. The bill requires that all Nigerians be covered by a health insurance plan and have access to basic health services, including routine immunizations. It also endeavors to address health inequities through a special fund for vulnerable persons, including pregnant women and children under the age of five [3]. Despite the bill, there are many significant barriers to improving healthcare access, including corruption; fragmented health insurance streams; issues with primary, secondary, and tertiary healthcare structures; inadequate monetary and human resources; and political interference in public health programs [3].

Historically, there has been little consensus on how to define and measure healthcare access [4]. In 2013, Levesque et al. systematically analyzed the existing conceptualizations of healthcare access toward the development of a holistic Conceptual Framework for Healthcare Access. Through this analysis, the researchers came to define healthcare access as “the opportunity to identify healthcare needs, to seek healthcare services, to reach, to obtain or use health care services and to actually have the need for services fulfilled” [4] (p. 8). The Framework has since been widely adopted by health system researchers [5]. Qualitative, quantitative, and mixed-method studies across populations and contexts have applied the framework, including the study of global trends of healthcare access in low- and middle-income countries (LMICs) [5] and is a strong framework for researchers and decision-makers to use when studying both supply and demand aspects of healthcare access and, for example, childhood immunization rate data in Nigeria.

Improving UHC through the domains of healthcare accessibility involves both the health system and the populations that utilize it [4]. The first dimension of the Framework for Healthcare Access [4], approachability, refers to how health services make themselves known and how users perceive their availability, which is shaped by health literacy, knowledge, and beliefs [4]. Nigerian literature has emphasized supply-side barriers and called on government and health systems to enhance accessibility [6,7], while population-focused studies highlight the influence of sociodemographic factors such as wealth, age, education, ethnicity, and geography on healthcare access [8,9,10,11,12]. Given the framework’s effectiveness in low- and middle-income countries (LMICs), it presents a valuable lens for examining healthcare access and approachability in Nigeria using immunization data.

Researchers have explored how perceptions of health information affect health behaviors in Nigeria. Mass media and internet communication technology (ICT) are commonly used to disseminate health information [13] and have been shown to improve health literacy and promote positive health behaviors [14,15,16]. However, limited access to these technologies remains a barrier. Studies link low immunization rates to a lack of information [17,18], and qualitative research suggests that improving health communication—such as using radio, the most accessible medium in some communities—can enhance coverage [18,19]. Despite this, only 5% of women and 13% of men regularly access traditional media, and internet usage remains low at 21% for women and 28% for men [20].

This study aimed to examine healthcare access in Nigeria by analyzing the relationship between childhood immunization rates, exposure to mass media and ICT, and sociodemographic factors using the 2021 Multiple Indicator Cluster Survey—Nigeria Integrated Community Survey (MICS-NICS) datasets [20]. Through quantitative analysis and geospatial mapping, the research explored regional disparities and applied Levesque et al.’s Framework for Healthcare Access [4], focusing on the approachability dimension to assess how access to health information influences immunization outcomes. The study was guided by two research questions:

1. AreNigerian mothers/caregivers of children with incomplete immunizations less likely to be exposed to mass media and ICT?
2. Do sociodemographic factors influence the relationship between media/ICT exposure and incomplete immunization?

## 2. Materials and Methods

### 2.1. Dataset and Population

This study involved the quantitative analysis of secondary data from the 2021 MICS-NICS [4]. MICS-NICS data in Nigeria are routinely collected through household surveys by trained enumerators with nationally representative selected households to gather information on health and other key indicators. The data used in this study were from the Individual Women and Child Under 5 surveys and were merged by IBM SPSS Statistics Version 29.0.2.0 software using “household ID” variables to link each child to their corresponding mother/caregiver. The target population of study was mothers in Nigeria of children aged 24–48 months (2–3 years) from a nationally representative sample (*n* = 12,533).

### 2.2. Dependent and Independent Variables

The dependent variable (DV) was childhood immunization status among 2- and 3-year-olds. The DV data were transformed into a binary value (Complete Immunizations = 0 and Incomplete Immunizations = 1) to align with the study’s focus on children not fully vaccinated (incomplete immunization) as per the recommended schedule. Previous literature has established using binary logistic regression to explore the determinants of immunization status in children [21,22,23,24,25]. For example, Ogundele et al. adopted an additive composite variable approach [24], and Ahmed et al. applied binary logistic regression [25] analyzing whether children had completed the required vaccination or otherwise. For this analysis, this required merging the “zero dose” and “incomplete” answers from the raw data in to a singular “incomplete” variable. The sociodemographic independent variables for this study were selected based on what was available in the existing MICS-NICS Nigeria 2021 datasets and which factors had been identified in previous literature to be related to healthcare access [8,9,10,11,12].

Media/ICT exposure variables were selected from data available in the datasets and categorized as individual and household sociodemographic factors or media access factors (see Table 1). Geographic identifiers—including area of residence, administrative regions, and geopolitical zone—were included to account for spatial variation in child immunization and report differences at multiple geographic scales. Missing data were handled using listwise deletion, which reduced the sample size by approximately 20%.

### 2.3. Statistical Analyses

First, the frequences of the DV and IVs were determined to understand the distribution of the variables, provide insight into variability within the data, and identify potential outliers. Next, a bivariate cross-tabulation analysis using a chi-square test was conducted to uncover potential statistical relationships between the DV and IVs. This step identified which IVs had significant correlational relationships to the DV (defined as *p*-values < 0.01). Finally, significant IVs identified from the cross-tabulation analysis were included in a linear logistic regression model analysis. This advanced analysis determined the predictive power of the IVs on the DV, while controlling for potential confounding or redundant factors. The threshold of *p*-value < 0.01 minimizes the risk of Type 1 errors. The confidence interval for the linear regression analysis was set at 95% to further ensure a high degree of reliability in parameter estimates.

### 2.4. Geospatial Mapping

The results from the bivariate and logistic regression analyses were input into a geographic information system (GIS). Thematic maps have been found to enrich the interpretation of regression analysis by presenting a visual spatial dimension to the statistical relationships [26,27,28], highlighting which regions should receive priority interventions [29].

## 3. Results

The results of the statistical analyses demonstrate the relationship between sociodemographic factors, media access, and healthcare access. The outputs are shown through the tables, summaries, and discussion below and conclude that while there was nuance to how the variables interact, the primary driver of how healthcare was accessed was region.

### 3.1. Frequency Analysis

The frequency distribution of respondents across the included sociodemographic variables is shown in Table 2 and demonstrates wide variance across the factors. Respondents were represented from all 37 regions, with the smallest proportion being from Bayelsa (0.9%) and the largest from Kano (7.4%). Across geopolitical zones, the highest proportion of respondents were from the northwest and northeast (32.2% and 17.1%, respectively) and over 63% of respondents were from rural areas. The education levels of mothers varied, with 40.6% of respondents having zero education, 27.1% having attended senior secondary education, and primary, junior secondary, and higher/tertiary trailing behind in proportion. Most mothers in the study fell between the ages of 20–44 years old, with only 1.9% being younger and 3.5% being older. The children of the respondents were equally represented by sex and a considerable proportion of the children had incomplete immunization statuses (73.8%). Very few respondents reported any kind of health insurance (only 2.7%). The wealth index showed that the largest number of respondents came from the poorest two quintiles (46.6% in total). The most prevalent ethnicities represented were Hausa (33%), Yoruba (12.7%), Igbo (11.9%), and “other” (23.1%).

Media and ICT access among the respondents was generally limited, as shown in Table 3. Notably, only 32.5% of respondents had ever read a newspaper or magazine, 35.8% had ever listened to the radio, and 8.1% of respondents had ever watched television. A substantial proportion of respondents had never accessed digital media (70.1%). Mobile phone ownership was highest at 43.5% of those sampled.

### 3.2. Bivariate Cross-Tabulation Analysis Using Chi-Square Test

The statistical results from the bivariate analysis between the immunization status of the child and the sociodemographic and media factors are presented in Table 4. All of the independent variables showed a statistically significant correlational relationship (*p* < 0.001) with the dependent variable except sex of the child (*p* = 0.362). Immunization rates were found to be higher among children from wealthier households in urban areas and if their mothers had more education and/or higher maternal age. Ethnicity was also shown to be associated with higher rates of incomplete immunizations; children from Fulani (85.3%), Hausa (84.7%), and Kanuri (83.3%) households were more likely to have incomplete immunizations than those from Igbo (53.7%) or Yoruba (60.2%) households.

Media access was significantly associated with immunization status. Children whose mothers had never been exposed to traditional or digital media sources were significantly more likely to have incomplete immunization status (78.8% incomplete coverage to 82.7% incomplete coverage). Children whose mothers did not own a mobile phone were also significantly more likely to have incomplete immunization status (84.2%) compared to those whose mothers did own a mobile phone (69.5%). However, despite the significant relationship between media/ICT access and immunization rates, children whose mothers did have media access were still generally likely to have incomplete immunization status (ranging from 55.3% to 71.1%). Finally, strong regional and geopolitical differences were noted in the bivariate analysis (*p* < 0.001). For example, Sokoto (97%), Bauchi (94.6%), and Zamfara (94.8%) exhibited the highest proportions of children with incomplete immunizations. Ebonyi (26.1%), Anambra (50.3%), and Lagos (50.0%) had the lowest proportions. The Imo region’s (73.4%) rates of incomplete immunization were closest to the total national average in Nigeria (73.8%).

**Figure 1:**
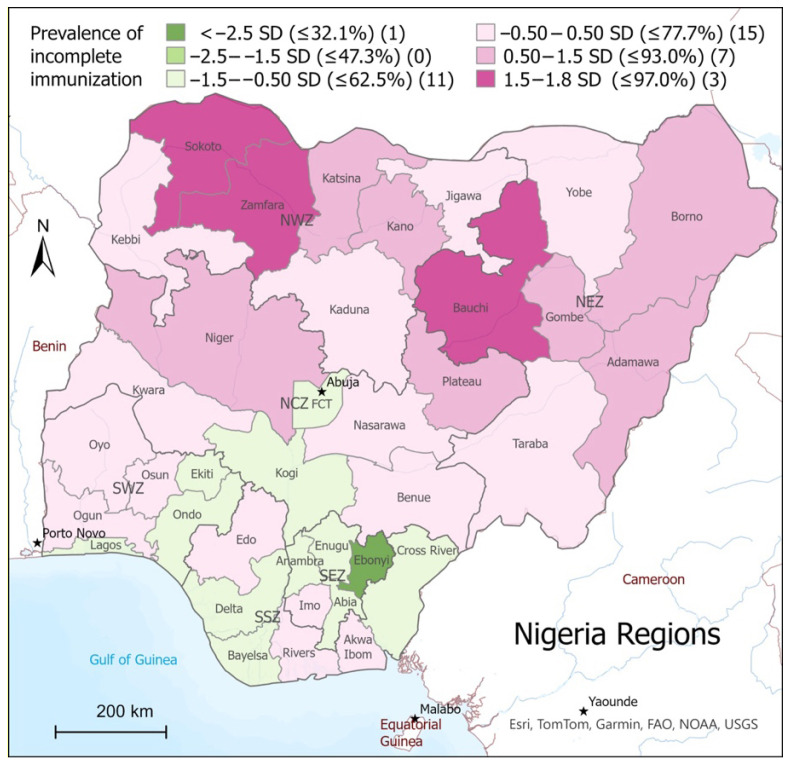
Prevalence of incomplete immunization by region in relation to the national mean.

Geospatial mapping (Figure 1) was used for the identification of clusters or regions with higher rates of incomplete immunizations compared to the national average. The map illustrates disparities by showing how each region’s rate of incomplete immunizations deviated from the national mean. Regions with rates within –0.5 and above, up to 1.8 standard deviations (SD) from the mean, are shaded in light pink, with darker shades of pink representing significantly higher rates of incomplete immunization. Regions that fell below –0.5 SD from the national mean are shaded in green, with the darker shades representing significantly lower rates of incomplete immunizations.

### 3.3. Linear Logistic Regression Model Analysis

To account for and discern between potentially confounding variables, logistic regression modeling was conducted, and adjusted odds ratios (AORs) were calculated (CI = 95%) using the significant IV (*p* < 0.01) identified in the bivariate cross-tabular analysis. The AORs represent the likelihood that a child has incomplete immunizations, adjusted for sociodemographic and media-access-related factors. The logistic regression model demonstrated acceptable fit to the data. The final model converged after six iterations, with parameter estimates stabilizing (change < 0.001). The −2 Log Likelihood value was 9461.603, indicating the model’s overall fit to the observed data. To assess the explanatory power of the model, pseudo R-squared values were examined. The Cox and Snell R-square was 0.138, and the Nagelkerke R-square was 0.208, suggesting that approximately 20.8% of the variance in the DV is explained by the predictors included in the model.

As shown in Table 5, the logistic regression analysis revealed several factors predictive of a child’s immunization status. These included region, mother’s age, education, and ethnicity, as well as mother’s TV use and mobile phone ownership. Higher levels of maternal education were strongly associated with lower incidence of incomplete vaccinations. Children whose mothers had senior secondary education (AOR = 0.649, *p* < 0.001) or higher/tertiary education (AOR = 0.573, *p* < 0.001) had significantly lower odds of having incomplete immunizations compared to those whose mothers had no formal education. Mobile phone ownership (AOR = 1.155, *p* = 0.034) was significantly associated with higher odds (15.5%) of incomplete immunizations. The strongest media factor was TV use (AOR = 0.63, *p* < 0.001), with those who had ever watched television having 36.4% lower odds of incomplete immunization for their children.

**Figure 2:**
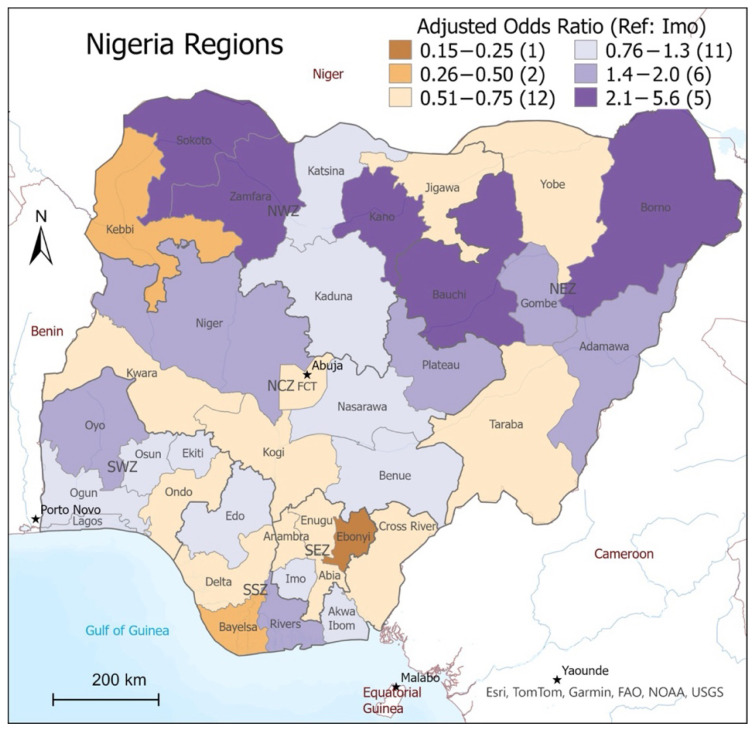
Adjusted odds ratios of incomplete immunizations by region.

Region had a highly predictive association with immunization status overall (*p* < 0.001), but the effect differed from region to region. The Imo region was found to be closest to the total national average, and so the model used the Imo region as a reference point to determine which states were more or less likely to have higher rates of incomplete immunizations, and geospatial mapping was used to visualize the AORs regionally (Figure 2). States colored light purple (AOR = 0.76–1.3) were the most likely to have immunization rates comparable to the reference region. The darker purple regions represent a higher adjusted odds of incomplete immunizations, and the orange represents a lower adjusted odds of incomplete immunizations, with the darkest orange being the lowest (AOR = 0.15–0.25). The map illustrates the finding from the regression model that Sokoto (AOR = 5.6, *p* < 0.001), Bauchi (AOR = 3.5, *p* < 0.001), Zamfara (AOR = 3.0, *p* < 0.001), Kano (AOR = 2.9, *p* = <0.001), and Borno (AOR = 2.4, *p* <0.001) had the highest odds of incomplete immunizations and that Kebbi (AOR = 0.32, *p* <0.001), Bayelsa (AOR = 0.28, *p* < 0.001), and Ebonyi (AOR = 0.15, *p* < 0.001) had the lowest in comparison to the national mean.

Overall, the results of the study demonstrate that the data are nationally representative, that region is the strongest sociodemographic predictive factor in immunization status for children, childhood immunization rates are influenced by access to media/ICT, and that disparities in both traditional and digital media access exist in Nigeria.

## 4. Discussion

This study explored the relationship between maternal media access and childhood immunization status by leveraging the approachability dimension of Levesque’s Conceptual Framework for Healthcare Access [4] as a guiding lens. This dimension of the framework connects how visible, transparent, and communicated a health system’s services are in relation to a population’s ability to recognize their healthcare needs and understand how to access services (i.e., health literacy).

In this context, maternal exposure to media, particularly television, appears to play a role in shaping health literacy and healthcare-seeking behavior. The analysis revealed significant associations between media access and immunization outcomes, but it is important to underscore that the study’s cross-sectional design precludes any causal inference. While maternal television viewing was positively associated with completed childhood immunizations, and mobile phone ownership was negatively associated, these relationships should be interpreted with caution. The observed associations may be influenced by unmeasured confounding variables, such as regional health infrastructure, cultural norms, or trust in health systems, which were not fully captured in the dataset or this analysis.

The contradictory finding regarding mobile phone ownership is particularly noteworthy. While mobile phones have been found to enhance access to health information [30,31], their negative association with immunization rates in this analysis suggests that more complex dynamics exist in this population. This could reflect disparities in digital literacy, the prevalence of misinformation, or differences in how mobile phones are used. Future research should explore these nuances through qualitative methods and consider disaggregating mobile phone use by function (e.g., SMS, voice calls, social media, internet use, health applications) to better understand its impact on health service approachability and health behaviors.

The mothers sampled in this study had lower rates of mobile ownership (43.5%) than the national household rate (87.6%), a finding that is aligned with previous literature that addresses how gendered digital disparities disproportionately impact women’s health; these studies caution that media/ICT health communication strategies risk worsening inequities if the digital divide is not addressed [32]. This may suggest that the specific medium of exposure and/or the strategies used in health communications may influence how information is perceived by populations and should be explored in future studies. This finding is aligned with previous literature that supports the use of mass media campaigns to influence health behaviors in LMICs [33], but that digital health technologies need to be accessible, affordable, and population- and context-specific to be effective [34].

Regional disparities in immunization coverage were also pronounced. Northern states such as Sokoto, Zamfara, Bauchi, Kano, and Borno had rates of incomplete immunization exceeding 90%, while southern states like Ebonyi and Lagos reported significantly lower rates. These patterns align with existing literature on geographic inequities in healthcare access, often rooted in infrastructural deficits, sociopolitical instability, and sociocultural norms [35]. However, the representativeness of the MICS-NICS data must be considered when interpreting these findings. The survey’s reliance on household responses in selected clusters, some of which had low response rates, raises concerns about external validity. Although supplementary households were added and weighted data were used to mitigate bias, the potential for underrepresentation of hard-to-reach populations remains.

From a statistical standpoint, the study did not include a formal assessment of multicollinearity, such as Variance Inflation Factor (VIF) calculations. This omission means that potential correlations among independent variables may not have been fully accounted for, potentially affecting the robustness of the regression models. Additionally, the use of listwise deletion to handle missing data may have introduced bias, particularly if missingness was not random. For example, respondents from rural or less educated backgrounds may have been more likely to have incomplete responses, leading to their exclusion and potentially skewing the results. Future research should consider more sophisticated approaches to missing data to ensure that vulnerable populations are adequately represented.

Another limitation is the exclusion of male caregivers from the analysis due to constraints in merging datasets within SPSS. This omission restricts the study’s ability to capture the full spectrum of family-level decision-making around immunization. Given that fathers and other caregivers may play influential roles in health decisions, future studies should aim to include these perspectives to provide a more comprehensive understanding.

The timing of data collection also warrants attention. The 2021 MICS-NICS survey was conducted during the early stages of the COVID-19 pandemic, a period marked by rapid shifts in health communication strategies and public health priorities [36]. The pandemic potentially influenced both media consumption patterns and attitudes toward vaccination, which may limit the generalizability of the findings to post-pandemic contexts. The impact of the pandemic on how health media and communications are perceived could be explored using qualitative measures by future researchers.

Looking ahead, the next iteration of the MICS-NICS survey may reflect evolving trends in media and ICT access. Repeating this analysis using updated data would allow for the longitudinal assessment of these dynamics. The continued application of Levesque et al.’s framework [4] and qualitative exploration into how different population groups use and perceive media in relation to health decisions will be instrumental in guiding both researchers and policy makers as they develop targeted strategies to improve immunization coverage and healthcare access in Nigeria.

### Study Strengths and Limitations

Study strengths include utilizing a large, nationally representative dataset, which allows for broad insights into maternal media access and childhood immunization patterns across Nigeria. The application of a valid conceptual framework provides a robust theoretical lens for interpreting healthcare access, and the integration of geospatial mapping adds depth to the analysis of regional disparities.

Several study limitations exist. First, the cross-sectional design limits the ability to draw causal conclusions. Second, the MICS-NICS dataset is thought to suffer from representativeness bias due to low response rates in certain clusters [37]. Despite efforts to mitigate this through supplementary sampling and data weighting [20], the possibility of representative bias remaining should be considered. Third, the exclusion of male caregivers restricts the analysis to maternal influences, omitting potentially important family-level determinants of immunization behavior. Fourth, the timing of data collection during the early COVID-19 pandemic may have influenced both media use and health-seeking behaviors, limiting the generalizability of findings to post-pandemic contexts. Fifth, the study did not formally assess multicollinearity among independent variables, including the geographic identifiers, which may affect the precision of regression models/coefficients. Finally, the use of listwise deletion to handle missing data may have further introduced representativeness bias against vulnerable groups.

## 5. Conclusions

This study underscores the role of maternal media and ICT access in health outcomes in Nigeria through the lens of health system approachability and health literacy. The findings reveal significant sociodemographic and regional disparities in media and ICT access, which carry important implications for equitable healthcare delivery.

To improve immunization coverage and advance progress toward universal health coverage and the Sustainable Development Goals, future research and policy efforts must prioritize inclusive, context-specific media and communication strategies. Addressing Nigeria’s digital divide is a necessary step toward enabling equitable, effective, and population-tailored health interventions and communications.

## Figures and Tables

**Table 1:** Descriptions of sociodemographic and media access independent variables.

Variable	Description
Sex of Child	Male or female
Health Insurance of Child	Whether or not the child has health insurance of any kind
Area of Residence	Urban or rural
Region	Which of 37 distinct regions a respondent was living
Geopolitical Zone	Which of 6 geopolitical zones a respondent was living
Mother’s Age	7 age ranges between 15 and 49
Mother’s Education	Highest level of education attended (but not necessarily completed)
Ethnicity of Household Head	Ethnicity of household head
Wealth Index Quintile	Household’s income status by quintile
Ever Read a Newspaper or Magazine ª	Whether a mother has ever read a newspaper or magazine
Ever Listened to Radio ª	Whether a mother has ever listened to the radio
Ever Watched Television ª	Whether a mother has ever watched television
Ever Used Internet ª	Whether a mother has ever used the internet
Own a Mobile Phone ª	Whether or not a mother owns a mobile phone

ª Variable converted into binary (yes/no) from original categorical dataset.

**Table 2:** Percentage of respondents by sociodemographic variables.

Variables	Frequency	Percent	Variables	Frequency	Percent
Region			Childhood Immunization Status		
Abia	201	1.6	Complete	3283	26.2
Adamawa	299	2.4	Incomplete	9250	73.8
Akwa Ibom	259	2.1	**Area**		
Anambra	318	2.5	Urban	4601	36.7
Bauchi	651	5.2	Rural	7933	63.3
Bayelsa	111	0.9	**Mother’s education ª**		
Benue	386	3.1	None	5093	40.6
Borno	367	2.9	Primary	1946	15.5
Cross River	192	1.5	Junior secondary	820	6.5
Delta	257	2.0	Senior secondary	3402	27.1
Ebonyi	188	1.5	Higher/tertiary	1270	10.1
Edo	204	1.6	**Mother’s Age ª**		
Ekiti	151	1.2	15–19	239	1.9
Enugu	217	1.7	20–24	1682	13.4
Gombe	260	2.1	25–29	2763	22.0
Jigawa	539	4.3	30–34	2442	19.5
Kaduna	562	4.5	35–39	1897	15.1
Kano	927	7.4	40–44	985	7.9
Katsina	761	6.1	45–49	437	3.5
Kebbi	413	3.3	**Health insurance ª**		
Kogi	229	1.8	With insurance	337	2.7
Kwara	198	1.6	Without insurance	12,167	97.1
Lagos	716	5.7	**Wealth index quintile ª**		
Nasarawa	180	1.4	Poorest	3029	24.2
Niger	395	3.2	Second	2813	22.4
Ogun	327	2.6	Middle	2440	19.5
Ondo	206	1.6	Fourth	2200	17.6
Osun	193	1.5	Richest	2050	16.4
Oyo	376	3.0	**Sex of Child**		
Plateau	300	2.4	Male	6263	50.0
Rivers	383	3.1	Female	6270	50.0
Sokoto	434	3.5	**Ethnicity of household head**		
Taraba	272	2.2	Hausa	4135	33.0
Yobe	290	2.3	Igbo	1496	11.9
Zamfara	401	3.2	Yoruba	1589	12.7
FCT	144	1.1	Fulani	1179	9.4
Imo	229	1.8	Kanuri	318	2.5
**Geopolitical Zone**			Ijaw	179	1.4
North Central	1831	14.6	Ibibio	252	2.0
North East	2138	17.1	Edo	164	1.3
North West	4037	32.2	Tiv	327	2.6
South East	1153	9.2	Other ethnicity	2893	23.1
South South	1405	11.2			
South West	1968	15.7			

ª Missing values not included.

**Table 3:** Percentage of respondents by media access variables.

Variables	Frequency	Percent
**Television ª**		
Have Never Watched TV	9424	75.2
Have Watched TV	1015	8.1
**Radio ª**		
Have Never Listened to Radio	5957	47.5
Have Listened to Radio	4488	35.8
**Newspaper/Magazine ª**		
Have Never Read Newspaper/Magazine	6363	50.8
Have Read Newspaper/Magazine	4075	32.5
**Internet Use (Ever) ª**		
Yes	1391	11.1
No	8780	70.1
**Own a Mobile Phone ª**		
Yes	5456	43.5
No	4983	39.8

ª Missing values not included.

**Table 4:** Bivariate analysis of the factors associated with immunization status of child.

Factors	Immunization Status of Child		Chi-Square *p*-Value
	Complete	Incomplete	
**Individual Factors**			
***Sex of Child***			0.362
Male	1663 (26.6%)	4600 (73.4%)	
Female	1620 (25.8%)	4650 (74.2%)	
***Health insurance***			<0.001
With insurance	131 (38.9%)	206 (61.1%)	
Without insurance	3149 (25.9%)	9019 (74.1%)	
**Household Factors**			
***Area of Residence***			<0.001
Urban	1670 (36.3%)	2930 (63.7%)	
Rural	1612 (20.3%)	6320 (79.7%)	
***Region***			<0.001
Abia	86 (43.0%)	114 (57.0%)	
Adamawa	43 (14.4%)	255 (85.6%)	
Akwa Ibom	82 (31.7%)	177 (68.3%)	
Anambra	158 (49.7%)	160 (50.3%)	
Bauchi	35 (5.4%)	615 (94.6%)	
Bayelsa	47 (42.3%)	64 (57.7%)	
Benue	109 (28.2%)	277 (71.8%)	
Borno	32 (8.7%)	335 (91.3%)	
Cross River	79 (40.9%)	114 (59.1%)	
Delta	103 (40.1%)	154 (59.9%)	
Ebonyi	139 (73.9%)	49 (26.1%)	
Edo	68 (33.3%)	136 (66.7%)	
Ekiti	59 (39.1%)	92 (60.9%)	
Enugu	103 (47.5%)	114 (52.5%)	
Gombe	28 (10.7%)	233 (89.3%)	
Imo	61 (26.6%)	168 (73.4%)	
Jigawa	125 (23.2%)	414 (76.8%)	
Kaduna	141 (25.1%)	420 (74.9%)	
Kano	79 (8.5%)	849 (91.5%)	
Katsina	136 (17.9%)	625 (82.1%)	
Kebbi	147 (35.7%)	265 (64.3%)	
Kogi	111 (48.5%)	118 (51.5%)	
Kwara	57 (28.9%)	140 (71.1%)	
Lagos	358 (50.0%)	358 (50.0%)	
Nasarawa	43 (23.9%)	137 (76.1%)	
Niger	47 (11.9%)	348 (88.1%)	
Ogun	87 (26.6%)	240 (73.4%)	
Ondo	86 (41.7%)	120 (58.3%)	
Osun	65 (33.7%)	128 (66.3%)	
Oyo	119 (31.6%)	257 (68.4%)	
Plateau	48 (16.0%)	252 (84.0%)	
Rivers	123 (32.1%)	260 (67.9%)	
Sokoto	13 (3.0%)	421 (97.0%)	
Taraba	90 (33.3%)	181 (66.8%)	
Yobe	96 (33.1%)	194 (66.9%)	
Zamfara	21 (5.2%)	380 (94.8%)	
FCT	56 (39.2%)	87 (60.8%)	
***Geopolitical Zone***			<0.001
North Central	470 (25.7%)	1361 (74.3%)	
North East	325 (15.2%)	1813 (84.8%)	
North West	663 (16.4%)	3374 (83.6%)	
South East	548 (47.5%)	605 (52.5%)	
South South	501 (35.7%)	904 (64.3%)	
South West	775 (39.4%)	1194 (60.6%)	
***Mother’s Age***			
15–19	39 (16.3%)	201 (83.8%)	<0.001
20–24	301 (17.9%)	1380 (82.1%)	
25–29	577 (20.9%)	2185 (79.1%)	
30–34	709 (29.0%)	1733 (71.0%)	
35–39	511 (26.9%)	1386 (73.1%)	
40–44	227 (23.0%)	758 (77.0%)	
*45–49*	88 (20.1%)	349 (79.9%)	
***Mother’s Education***			<0.001
None	759 (14.9%)	4334 (85.1%)	
Primary	482 (24.8%)	1464 (75.2%)	
Junior secondary	195 (23.8%)	626 (76.2%)	
Senior secondary	1290 (37.9%)	2112 (62.1%)	
Higher/tertiary	557 (43.9%)	713 (56.1%)	
***Ethnicity of Household Head***			<0.001
Hausa	634 (15.3%)	3501 (84.7%)	
Igbo	692 (46.3%)	804 (53.7%)	
Yoruba	633 (39.8%)	957 (60.2%)	
Fulani	173 (14.7%)	1005 (85.3%)	
Kanuri	53 (16.7%)	265 (83.3%)	
Tiv	90 (27.5%)	237 (72.5%)	
Ijaw	65 (36.1%)	115 (63.9%)	
Ibibio	90 (35.7%)	162 (64.3%)	
Edo	65 (39.6%)	99 (60.4%)	
Other ethnicity	789 (27.3%)	2104 (72.7%)	
***Wealth Index Quintile***			<0.001
Poorest	475 (15.7%)	2554 (84.3%)	
Second	539 (19.2%)	2274 (80.8%)	
Middle	645 (26.4%)	1795 (73.6%)	
Fourth	704 (32.0%)	1496 (68.0%)	
Richest	918 (44.8%)	1132 (55.2%)	
**Mother’s Media Access Variables**			
***Ever Read a Newspaper/Magazine***			<0.001
Yes	1345 (33.0%)	2729 (67.0%)	
No	1103 (17.3%)	5260 (82.7%)	
***Ever Listened to Radio***			<0.001
Yes	1297 (28.9%)	3191 (71.1%)	
No	1156 (19.4%)	4801 (80.6%)	
***Ever Watched Television***			<0.001
Yes	454 (44.7%)	561 (55.3%)	
No	1995 (21.2%)	7429 (78.8%)	
***Ever Used Internet***			<0.001
Yes	545 (39.2%)	846 (60.8)	
No	1810 (20.6%)	6970 (79.4%)	
***Own a Mobile Phone***			<0.001
Yes	1664 (30.5%)	3792 (69.5%)	
No	789 (15.8%)	4194 (84.2%)	

**Table 5:** Linear regression model of sociodemographic and media factors on immunization rates.

Factors	B	Sig.	AOR	95% CI for AOR	
				Lower	Upper
**Individual Factors**					
***Health insurance***					
With insurance (ref)					
Without insurance	−0.251	0.119	0.778	0.567	1.067
**Household Factors**					
***Area***					
Urban (ref)					
Rural	0.077	0.317	1.080	0.929	1.255
***Region***					
Imo (ref)		<0.001 *			
Abia	−0.481	0.034 *	0.618	0.396	0.964
Adamawa	0.685	0.024 *	1.984	1.092	3.602
Akwa Ibom	0.004	0.990	1.004	0.568	1.774
Anambra	−0.677	0.001 *	0.508	0.339	0.762
Bauchi	1.224	<0.001 *	3.400	1.906	6.063
Bayelsa	−1.263	<0.001 *	0.283	0.135	0.592
Benue	−0.085	0.798	0.918	0.478	1.762
Borno	0.864	0.006 *	2.372	1.277	4.404
Cross River	−0.651	0.017 *	0.522	0.305	0.892
Delta	−0.546	0.040 *	0.580	0.344	0.976
Ebonyi	−1.882	<0.001 *	0.152	0.092	0.251
Edo	−0.137	0.646	0.872	0.485	1.567
Ekiti	−0.251	0.390	0.778	0.438	1.380
Enugu	−0.393	0.097	0.675	0.425	1.074
Gombe	0.654	0.037 *	1.924	1.039	3.564
Jigawa	−0.497	0.065	0.608	0.358	1.032
Kaduna	0.180	0.498	1.198	0.710	2.019
Kano	1.060	<0.001 *	2.885	1.695	4.913
Katsina	−0.090	0.732	0.914	0.545	1.532
Kebbi	−1.126	<0.001 *	0.324	0.193	0.545
Kogi	−0.550	0.050	0.577	0.329	1.012
Kwara	−0.686	0.025 *	0.504	0.276	0.918
Lagos	−0.154	0.51	0.857	0.543	1.355
Nasarawa	0.200	0.530	1.222	0.654	2.282
Niger	0.692	0.013 *	1.998	1.154	3.457
Ogun	0.197	0.465	1.217	0.719	2.062
Ondo	−0.575	0.039 *	0.563	0.326	0.971
Osun	−0.188	0.516	0.829	0.471	1.460
Oyo	0.401	0.150	1.493	0.865	2.577
Plateau	0.635	0.032 *	1.887	1.055	3.376
Rivers	0.482	0.077	1.619	0.949	2.763
Sokoto	1.727	<0.001 *	5.626	2.719	11.644
Taraba	−0.496	0.074	0.609	0.353	1.050
Yobe	−0.479	0.099	0.619	0.350	1.094
Zamfara	1.105	<0.001 *	3.019	1.574	5.790
FCT	−0.419	0.154	0.658	0.370	1.169
***Age***					
15–19 (ref)		<0.001 *			
20–24	0.100	0.622	1.105	0.743	1.642
25–29	0.009	0.963	1.009	0.685	1.486
30–34	−0.293	0.139	0.746	0.506	1.100
35–39	−0.046	0.817	0.955	0.644	1.416
40–44	−0.032	0.879	0.969	0.643	1.459
45–49	−0.011	0.961	0.989	0.631	1.551
***Mother’s education***					
None (ref)		<0.001 *			
Primary	−0.193	0.033 *	0.824	0.690	0.984
Junior secondary	−0.213	0.075	0.809	0.640	1.022
Senior secondary	−0.432	<0.001 *	0.649	0.540	0.780
Higher/tertiary	−0.558	<0.001 *	0.573	0.447	0.734
***Ethnicity of household head***					
Tiv (ref)		0.036 *			
Hausa	0.092	0.749	1.096	0.624	1.925
Igbo	−0.142	0.642	0.867	0.476	1.580
Yoruba	−0.314	0.287	0.731	0.410	1.302
Fulani	−0.198	0.503	0.820	0.459	1.465
Kanuri	−0.268	0.428	0.765	0.394	1.485
Ijaw	0.282	0.467	1.326	0.620	2.837
Ibibio	−0.368	0.272	0.692	0.359	1.334
Edo	−0.417	0.248	0.659	0.325	1.336
Other ethnicity	−0.165	0.544	0.848	0.498	1.445
***Wealth index quintile***					
Poorest (ref)		0.439			
Second	−0.088	0.303	0.915	0.774	1.083
Middle	−0.121	0.218	0.886	0.731	1.074
Fourth	−0.190	0.108	0.827	0.656	1.043
Richest	−0.269	0.056	0.764	0.580	1.007
**Mothers’ Media Access Variables**					
***Ever Read a Newspaper/Magazine***					
No					
Yes	−0.050	0.520	0.951	0.817	1.108
***Ever Listened to Radio***					
No					
Yes	0.031	0.631	1.031	0.909	1.170
***Ever Watched Television***					
No					
Yes	−0.452	<0.001 *	0.636	0.529	0.765
***Ever Used Internet***					
No					
Yes	0.101	0.223	1.107	0.940	1.302
***Own a Mobile Phone***					
Yes					
No	0.144	0.034 *	1.155	1.011	1.320

* p < 0.05.

## Data Availability

The raw data supporting the conclusions of this article will be made available by the authors on request.
